# A Novel Approach to Optimize the Rheology and Buildability of 3D-Printed Magnesium Phosphate Cement Composites Using Carbonated Recycled Aggregate

**DOI:** 10.3390/ma19061060

**Published:** 2026-03-11

**Authors:** Mingxu Chen, Xingyu Qu, Yilin Wang, Xingang Xu, Xuelin Liu, Heyang Wu, Qiuyi Li

**Affiliations:** 1 College of Civil Engineering & Architecture, Qingdao Agricultural University, Qingdao 266109, China; chenmx@qau.edu.cn (M.C.); xyqu2002@163.com (X.Q.); 19236131816@163.com (Y.W.); xuelinl0809@163.com (X.L.); 2 State Key Laboratory of Silicate Materials for Architectures, Wuhan University of Technology, Wuhan 430070, China; xuxg123@foxmail.com

**Keywords:** magnesium phosphate cement, recycled aggregate, rheological properties, 3D printing, structural deformation

## Abstract

Controlling structural deformation is essential for the structural stability of 3D-printed cement composites. In this paper, carbonated recycled aggregate (CRA) was incorporated into 3D-printed magnesium phosphate cement composites (MPCCs) to control rheology and improve buildability. Experimental results show that CRA increased the static yield stress from 2210.96 to 6238.18 Pa and the storage modulus. When the incorporation of CRA was more than 15%, the phase angle was less than 45°, indicating predominantly solid-like behavior. Additionally, due to the higher porosity, the compressive strength and flexural strength of 3D-printed MPCCs decrease with the increasing CRA content; however, the decline tendency becomes significantly more pronounced when CRA content exceeds 10%. Structural deformation decreased from 14.39% to 6.91%, attributed to the rough surface of CRA, which promotes more uniform stress transfer during stacking. This study demonstrates a simple upcycling route that improves the printing stability and sustainability of 3D-printed magnesium phosphate cement composites (MPCCs).

## 1. Introduction

3D concrete printing (3DCP) imposes stricter rheological requirements than conventional casting, because materials must be pumpable, extrudable, and sufficiently shape-stable after deposition [1,2,3]. Consequently, controlling yield stress, viscosity, and early viscoelasticity is critical to limit geometric distortion and layer collapse [4,5]. In traditional concrete casting, these properties are primarily controlled by adjusting the mix ratio; however, in 3D printing, due to the layer-by-layer accumulation process, additional challenges arise [6,7]. Precise control of the flowability of material is necessary to prevent issues such as collapse or poor interlayer bonding during printing [8]. The accurate regulation of material flowability and post-deposition structural recovery is essential to achieve a stable printing process and high interlayer formation quality [9,10].

Recycled aggregate (RA) typically carries adhered mortar and exhibits rough, porous surfaces that reduce slump in conventional concretes [11]; however, these natures are advantageous for 3DCP because of the increased static yield stress and buildability [12,13]. Carbonating RA further refines the surface with calcite, densifies micropores, and enhances particle interlocking, which is expected to reinforce structural build-up during printing [14,15,16]. This modification is particularly important for 3DCP, as materials need to maintain high strength and structural stability during the printing process to ensure the quality of printed specimens [17,18]. Sun et al. studied the impact of recycled aggregates on 3D-printed mortars and found that the use of carbonated recycled aggregates significantly improved printability and mechanical properties [19]. Tong et al. assessed the performance of 3D-printed concrete incorporating recycled aggregates, indicating that the use of carbonated recycled aggregates enhanced the structural performance and reduced deformation during the printing process [20].

Magnesium phosphate cement (MPC) offers rapid set, high early strength, and strong adhesion, making it attractive for 3D printing [21]. The rapid setting feature of MPC is particularly advantageous for reducing the time required for the printing process, enabling fast construction without compromising material strength [22]. In addition, MPC’s excellent bonding properties help enhance interlayer bonding during printing, improving the structural integrity of the printed object. Zhao et al. studied the application of MPC in 3D printing and noted that it quickly solidifies during the printing process, providing strong interlayer bonding and structural stability [23]. Yet the combined use of CRA in 3D-printed MPC composites (MPCCs) and its systematic effects on yield stress, viscoelasticity, and structural deformation remain underexplored.

While the use of RA in 3D-printing is becoming increasingly popular, this study introduces an innovative approach by incorporating CRA into MPCCs. The carbonation process significantly enhances the buildability and dimensional stability of 3D-printed structures, offering a novel solution to the challenges commonly encountered with traditional 3D printing materials.

Therefore, this study incorporated CRA (0–20 wt.%) into 3D-printed MPCCs and quantified its effects on the static/dynamic yield stress, viscoelastic moduli, phase angle, and structural deformation. In this study, a direct correlation between rheological parameters and deformation was established and attributed to the improvements to CRA-induced microstructural refinement and particle interlocking.

## 2. Materials and Methods

### 2.1. Raw Materials

**Figure 1:**
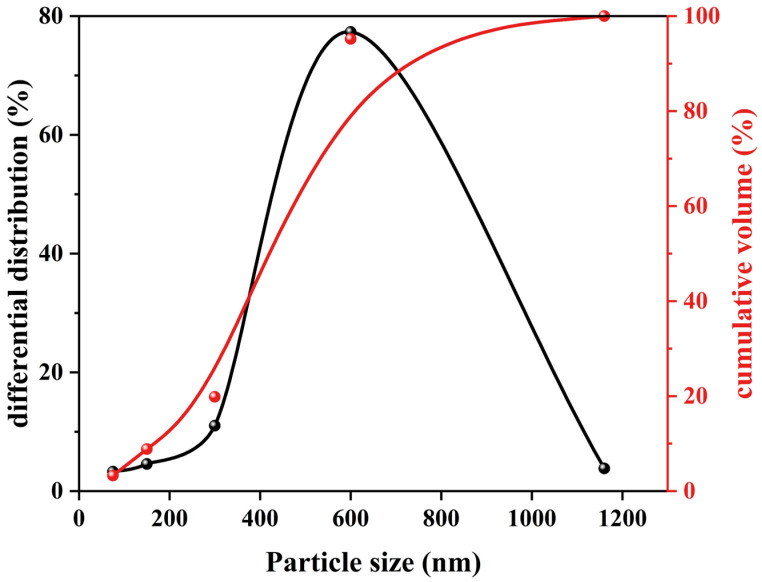
Particle size distribution of CRA.

**Figure 2:**
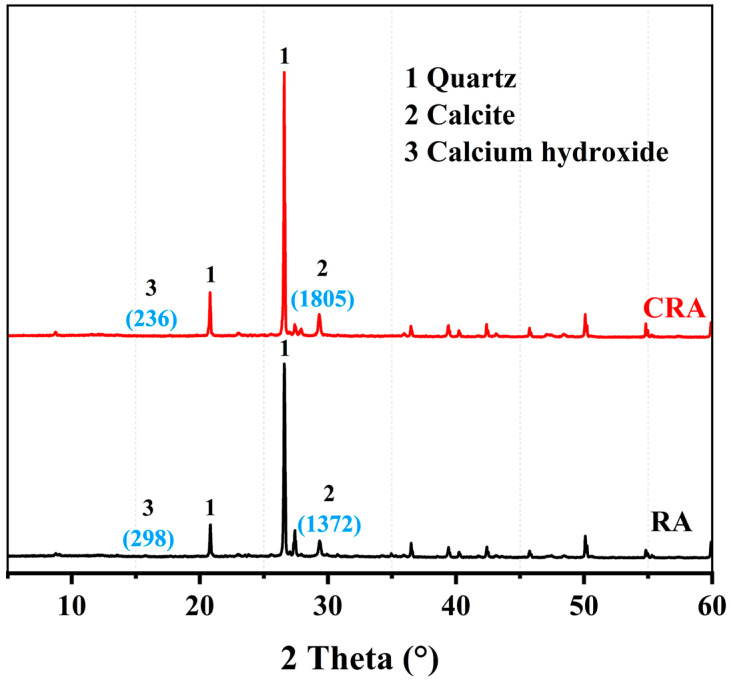
XRD pattern images of RA and CRA.

The studied 3D-printed MPCCs comprised four components: dead-burned MgO (Liaoning Tino Magnesium Industry Group Co., Ltd., Yingkou, China), silica fume (SF, Gongyi Yuanheng Water Purification Material Factory, Gongyi, China), slag (Lingshou Zhanteng Mineral Products Processing Plant, Shijiazhuang, China), and 100-mesh quartz sand (Gongyi Hengxin Filter Material Factory, Gongyi, China). CRA was obtained through the accelerated carbonation treatment for 3 days using a fully automated carbonation apparatus (HYT-THX-9, Beijing Hangjian Huaye Technology Development Co., Ltd., Beijing, China) on construction waste-derived aggregates with particle sizes below 4.75 mm, which were processed through crushing and sieving of demolition waste. Figure 1 shows the differential distribution and cumulative volume of CRA. Figure 2 presents the XRD patterns of the RA and CRA. The CRA exhibits a stronger intensity of the calcite diffraction and a weaker intensity of the Ca(OH)_2_ diffraction compared to the RA, which is likely due to the carbonation process, where Ca(OH)_2_ reacts with CO_2_ to form calcium carbonate.

### 2.2. Preparation Procedure

Mix proportions are listed in Table 1. In this study, the preparation procedure is as follows: Firstly, dry components (MgO, KH_2_PO_4_, silica fume, slag powder, and quartz sand) were dry-mixed for 2 min; then, CRA was added and dry-mixed for 2 min; finally, water was added and mixed for 2 min. Pastes were extruded through a 3D-printing nozzle to fabricate specimens.

### 2.3. Test Methods

All specimens were cured in a controlled environment at 20 °C and 95% relative humidity for 7 d to ensure consistent hydration and uniform properties across all tests. These conditions were maintained throughout the testing period to guarantee reproducibility and reliable evaluation of the rheological and mechanical properties.

#### 2.3.1. Rheological Properties

Rheological and viscoelastic properties of the pastes were systematically characterized using a rotary rheometer (HAAKE Mars 40, ThermoFisher, Waltham, MA, USA). Prior to testing, the pastes were pre-sheared at a constant shear rate of 100 s^−1^ for 60 s. Following pre-shearing, the samples were allowed to rest for 4 min to ensure full recovery of the microstructure. Static yield stress was subsequently determined by applying a low shear rate of 0.1 s^−1^ for 60 s. Flow curves were then obtained by incrementally increasing the shear rate from 0.1 to 100 s^−1^, and the resulting data were fitted using the Bingham model to extract the dynamic yield stress and plastic viscosity. Additionally, oscillatory shear measurements were performed at a fixed frequency of 1 Hz with a stress sweep ranging from 0.1 to 3000 Pa to determine the storage modulus (G′), loss modulus (G″), and phase angle (δ). All experiments were conducted in triplicate to ensure reproducibility and statistical reliability.

#### 2.3.2. Three-Stage Curve

Thixotropy is defined as a time-dependent and reversible decrease in viscosity under shear, followed by viscosity recovery once the shear is removed. The testing protocols were conducted in which the sample was first pre-sheared at 50 s^−1^ for 60 s and allowed to rest for 120 s to establish a reference structure, followed by shearing at 100 s^−1^ for 60 s to induce structural breakdown, and finally sheared at 0.1 s^−1^ for 120 s to monitor viscosity recovery.

#### 2.3.3. Mechanical Properties

The mechanical properties of the 3D-printed specimens were evaluated using a universal testing machine (MTS, Eden Prairie, MN, USA). Compressive and flexural strength tests were performed on cured samples after 7 days of curing at 20 °C and 95% relative humidity. Specimens were loaded at a constant rate of 0.3 kN/s within a range of 10–300 kN, and the strength was calculated based on the maximum applied load and the specimen dimensions. At least three specimens per mix were tested to ensure reliability. For each mix ratio, three specimens were tested to ensure repeatability and reliability of the results.

#### 2.3.4. Porosity

The porosity of the samples was measured using an automatic mercury porosimeter (AutoPore V 9600, Micromeritics Instrument Corporation, Norcross, GA, USA), covering a pore size range from 5 to 400 nm.

#### 2.3.5. Structural Deformation

In 3D-printed MPCCs, the calculation of structural deformation is an important step to evaluate the accuracy and stability of the printed specimens. Structural deformation refers to the dimensional changes that occur during or after the 3D-printing process, which may result from material shrinkage, internal stresses, or environmental factors such as curing conditions. Deformation is calculated using the following formula: $D\;=\;\frac{\left|L\;-\;L_{0}\right|}{{3L}_{0}}+\frac{\left|W\;-\;W_{0}\right|}{{3W}_{0}}+\frac{\left|H\;-\;H_{0}\right|}{{3H}_{0}}\;\times \;100\%$. Where D is the structural deformation; W_0_, L_0_, and H_0_ represent the size of the sample; and W, L, and H represent the maximum size of the printed specimen.

#### 2.3.6. Scanning Electron Microscope

The microstructures of the 3D-printed samples were observed using a scanning electron microscope (SEM) (Hitachi Regulus, 8100, Tokyo, Japan). Prior to imaging, the samples were immersed in isopropanol to exchange free water and subsequently dried at 45 °C for 24 h.

## 3. Results and Discussion

### 3.1. Yield Stress

#### 3.1.1. Static Yield Stress

**Figure 3:**
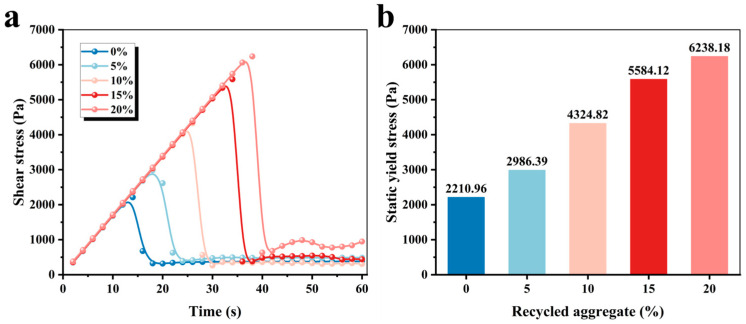
Effect of CRA content on the yield stress of 3D-printed MPCCs: (**a**) shear stress changes with time; (**b**) static yield stress.

The static yield stress is regarded as the ultimate stress before the microstructure is destroyed in the flocculation state, which is closely linked to time and is effective in monitoring the structural build-up of cementitious materials [24]. Figure 3 exhibits the effect of CRA content on the yield stress and apparent viscosity in 3D-printed MPCCs. Figure 3a shows that the shear stress initially increases and then decreases to a stabilized level. With the increase in CRA content, the time at which the peak shear stress occurs shifts progressively later, and the peak shear stress is regarded as the static yield stress. Figure 3b exhibits the variation in static yield stress of 3D-printed MPCCs with CRA content. The static yield stress increases from 2210.96 Pa to 6238.18 Pa with the increase in CRA content. The reason is that high water absorption and the rough surface of CRA will hinder the flow of the mixture and increase the yield stress.

#### 3.1.2. Dynamic Yield Stress

**Figure 4:**
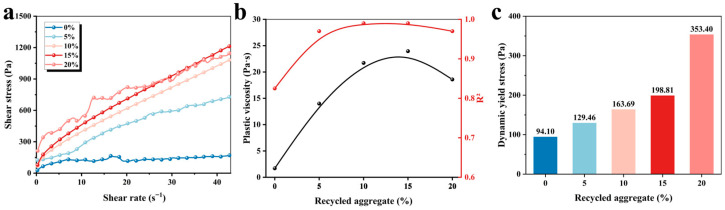
Effect of CRA content on the yield stress of 3D-printed MPCCs: (**a**) shear stress changes with variable shear rate; (**b**) plastic viscosity and R^2^; and (**c**) dynamic yield stress.

The dynamic yield stress, which reflects the ability of materials to resist flow under oscillatory conditions, plays a crucial role in determining the printability and structural stability of 3D-printed cementitious materials during the printing process [25,26]. Figure 4a demonstrates the shear stress changes with variable shear rates, indicating that the shear stress increases with the increased shear rate. The plastic viscosity and dynamic yield stress of 3D-printed MPCCs were calculated based on the Bingham model. Figure 4b shows the linear fitting with R^2^ > 0.95, indicating a strong correlation between the experimental and the fitted values. Figure 4c illustrates a clear positive correlation between the CRA content and dynamic yield stress. The dynamic yield stress increases from 94.10 Pa to 353.40 Pa as the CRA content rises from 0% to 20%, indicating that higher CRA content enhances the flow resistance, potentially due to increased interparticle friction.

### 3.2. Thixotropy

**Figure 5:**
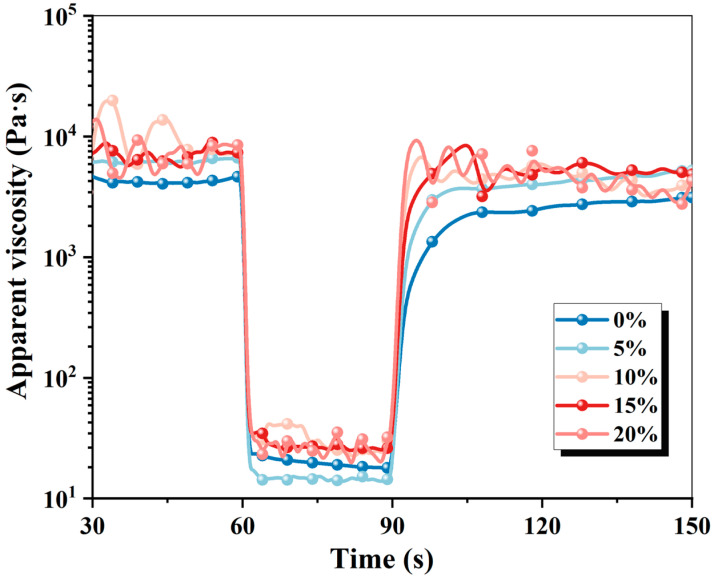
Apparent viscosity variation with time based on the three-stage curves.

Figure 5 illustrates the recovery degree and apparent viscosity of 3D-printed MPCCs changes with time based on the three-stage curves. As the shear stress increases from 0.1 to 100 s^−1^, the apparent viscosity of the paste decreases, indicating that higher shear stress promotes paste flow and reduces viscosity. When shear stress is removed, the paste structure gradually recovers to its initial state, leading to the apparent viscosity increasing. As the CRA content increases, the recovery degree of the paste improves, indicating that the addition of CRA enhances the ability to recover after structural deformation, which helps improve the structure stability in 3D-printing applications.

### 3.3. Viscoelasticity

**Figure 6:**
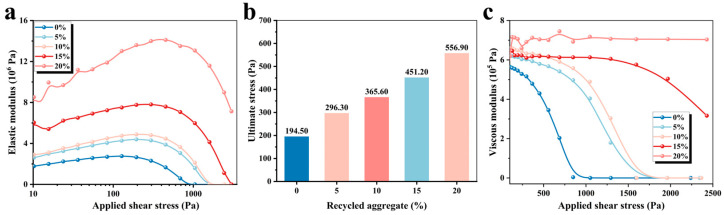
Effect of CRA content on the yield stress of 3D-printed MPCCs: (**a**) elastic modulus; (**b**) ultimate stress; and (**c**) viscous modulus.

The elastic and viscous moduli represent the ability of the material to store and consume energy during deformation, which can characterize the hardness of 3D-printed paste. Figure 6 shows the changes in the viscoelastic modulus and phase angle of 3D-printed MPCCs with shear stress. Figure 6a demonstrates that the elastic modulus of 3D-printed MPCCs changes with the applied shear stress, and the increased CRA content leads to a higher elastic modulus, suggesting that the CRA can raise the early stiffness of 3D-printed paste. Shear stress scanning tests the elastic and viscous behavior of materials through a linear increase in shear stress to determine the response of the microstructure. As shown in Figure 6b, as the CRA content increases from 0% to 20%, the limit stress in the linear viscoelastic region increases from 194.5 Pa to 556.9 Pa. Figure 6c shows that the viscous modulus decreases with the increasing content of CRA, except for the high CRA content.

**Figure 7:**
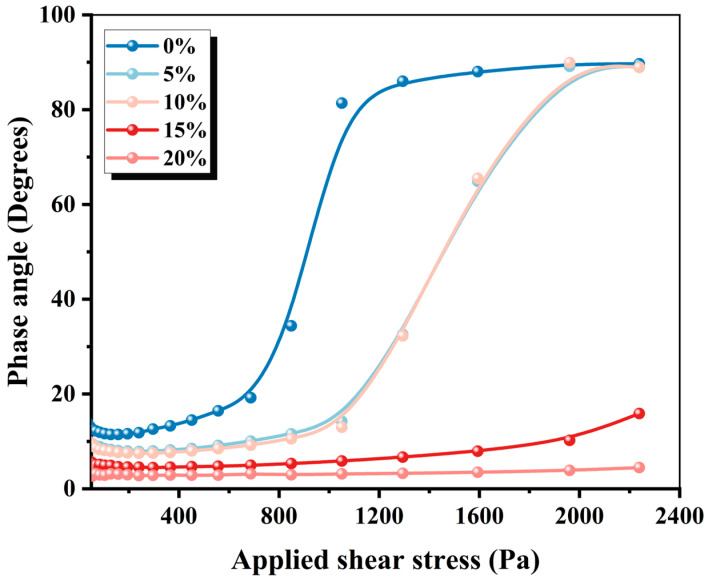
Phase angle of 3D-printed MPCCs.

**Figure 8:**
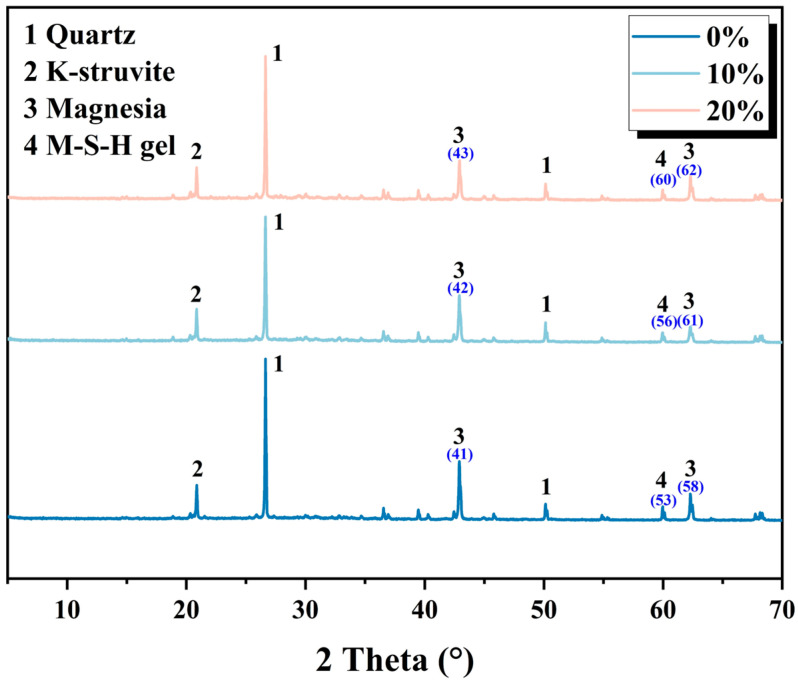
XRD patterns of 3D-printed MPCCs.

Moreover, the relationship between the elastic modulus and the viscous modulus can be expressed by the phase angle. Figure 7 demonstrates that the phase angle is always less than 45° as the contents of CRA increase to above 15%, indicating that the paste state of MPCCs is solid-like and stiff enough. To further elucidate the underlying mechanisms of the observed viscoelastic behavior, X-ray diffraction (XRD) analysis was conducted. Figure 8 analyzes the phase transformation mechanism of 3D-printed MPCCs using X-ray diffraction. The results indicate that CRA has a significant effect on the mineral composition and hydration products of 3D-printed MPCCs. As the CRA content increases, the mineral phases in 3D-printed MPCCs exhibit varying degrees of change, with characteristic peaks of M-S-H gel and Magnesia increasing with the CRA content. This results in a smaller phase angle, exhibiting quasi-solid-state behavior.

### 3.4. Mechanical Strength and Porosity

**Figure 9:**
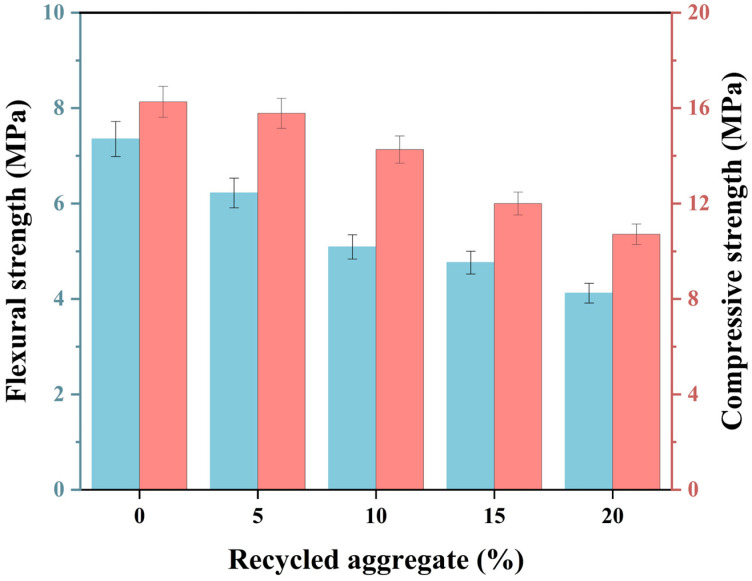
The compressive and flexural strength of 3D-printed MPCCs with different contents of CRA.

**Figure 10:**
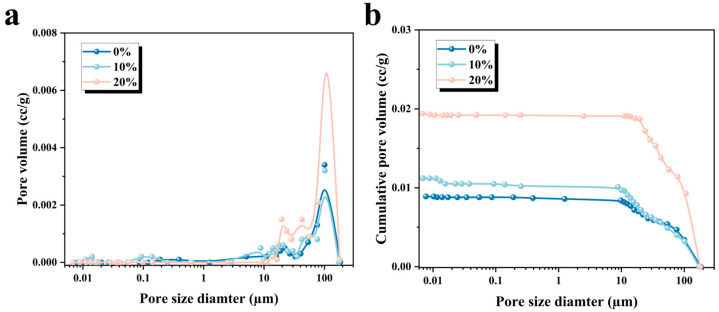
Pore structure analysis of 3D-printed MPCCs: (**a**) pore size distribution and (**b**) cumulative pore volume.

Mechanical strength is an essential criterion for evaluating the practical applicability of 3D-printed MPCCs. Figure 9 illustrates the flexural and compressive strength of 3D-printed MPCCs with varying CRA content. As shown, both the flexural and compressive strengths exhibit a decreasing trend when the CRA content reaches 20%, with the flexural strength dropping from 7.35 to 4.12 MPa and the compressive strength decreasing from 16.26 to 10.71 MPa. This decline may be attributed to the internal pore structure of the 3D-printed MPCCs. Mechanical strength is often influenced by the distribution and cumulative size of the pores within the hardened paste. Figure 10a,b present an analysis of the pore structure of the 3D-printed MPCCs. The results show that the materials exhibit a more porous structure, with a higher cumulative pore size as the CRA content increases from 0% to 20%. Consequently, the incorporation of CRA increases the porosity of the 3D-printed MPCCs, leading to a reduction in material density. However, the decrease in density is relatively small, and the impact on mechanical properties is minimal. Increased porosity improves printability and buildability, making the material suitable for non-structural applications. For structural uses, the CRA content of 10–15% strikes the best balance, maintaining strength while enhancing rheological performance, making it suitable for both structural and non-structural applications.

### 3.5. Structural Deformation

**Figure 11:**
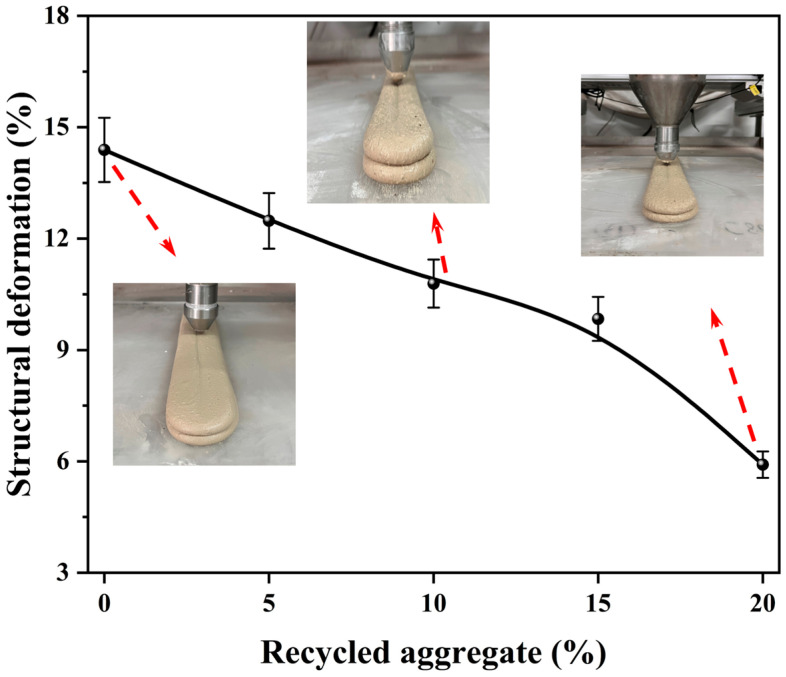
Macrograph and structure deformation of 3D-printed MPCCs.

**Figure 12:**
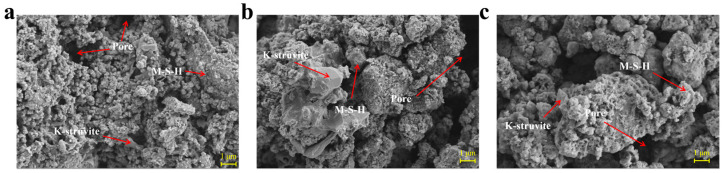
SEM results of (**a**) the control mixture; (**b**) mixture with 10% CRA content; and (**c**) mixture with 20% CRA content.

Structural deformation is one of the most important indicators for evaluating the printing accuracy and stability of 3D-printed cementitious materials [27]. Figure 11 displays the macroscopic images and structural deformation of 3D-printed MPCCs with different CRA contents. The macroscopic images of the printed samples demonstrate the influence of CRA content on the buildability of MPCCs. As the proportion of CRA increases, the structural deformation decreases. Increasing the CRA content from 0% to 20 wt.% reduces structural deformation from 14.39% to 6.91%. The incorporation of CRA effectively reduced structural deformation in 3D-printed MPCCs and enhanced structural stability, which may be related to rheological behavior. The results of this study are consistent with Sun et al. [19], which demonstrated that carbonated recycled aggregates enhance the printability of 3D-printed mortars. SEM observations further reveal the microstructural basis for this improvement. Figure 12 shows the SEM images of 3D-printed MPCCs with 0%, 10%, and 20% CRA. The surface of CRA promotes the nucleation and uniform distribution of hydration products, thereby reducing shape deformation. Consistent with the findings of Zhao et al. [23] on the effects of recycled aggregates in 3D-printed materials, this study further demonstrates that carbonation enhances the interfacial bonding between particles, thereby improving the overall structural integrity.

**Figure 13:**
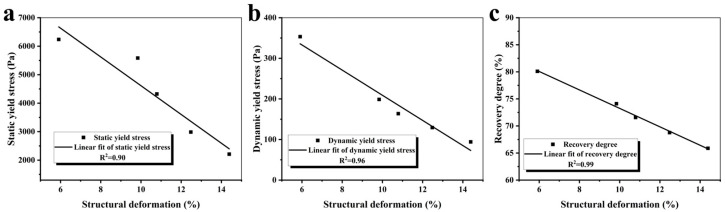
Correlation of thixotropy recovery rate, static/dynamic yield stress, and structural deformation. Correlation between structural deformation and (**a**) static yield stress; (**b**) dynamic yield stress; (**c**) thixotropy recovery rate.

Figure 13 shows the correlation of the thixotropy recovery rate, static/dynamic yield stress, and structure deformation. As shown in Figure 13, structural deformation correlates inversely with both the thixotropy recovery rate and static/dynamic yield stress, linking rheological build-up to print dimensional stability. The strengthened CRA–matrix interface facilitates more uniform stress transfer between layers, consistent with the reduced deformation.

## 4. Conclusions

In this study, a carbonated recycled aggregate (CRA) was incorporated into 3D-printable MPCCs, and their rheological behavior was optimized by regulating oscillatory shear and rotational shear scanning protocols. This strategy enabled a simultaneous improvement in extrudability, structural build-up stability, and shape retention. The application of CRA demonstrates significant potential for the performance regulation and sustainable development of 3D-printed MPCCs. The main conclusions are summarized as follows:

(1) The incorporation of CRA significantly increased the yield stress and improved the thixotropic behavior of 3D-printed MPCCs. As the CRA content increases, both static and dynamic yield stress increase, indicating a markedly enhanced resistance to flow and improved structural rebuilding capability.
(2) With the incorporation of CRA, the elastic modulus of 3D-printed MPCCs was significantly increased, accompanied by a pronounced reduction in the phase angle. This viscoelastic modification is beneficial for enhancing shape retention after extrusion and improving interlayer structural stability during the printing process.
(3) As the CRA content increased to 20 wt.%, the structural deformation of 3D-printed MPCCs during layer-by-layer stacking decreased from 14.39% to 6.91%, representing a reduction of 7.48%, thereby substantially improving the dimensional stability and buildability of the printed structures.
(4) A high CRA content exerted a certain adverse effect on the mechanical strength of 3D-printed MPCCs, which is mainly attributed to the increased porosity and the reduced effective hydration rate.

## Figures and Tables

**Table 1:** Mix proportion of 3D-printed MPCCs (% by mass).

MgO	KH_2_PO_4_	Silica Fume	Slag	Water	Quartz Sand	CRA
50	16.7	16.7	16.7	16	41.7	0
50	16.7	16.7	16.7	16	41.7	5
50	16.7	16.7	16.7	16	41.7	10
50	16.7	16.7	16.7	16	41.7	15
50	16.7	16.7	16.7	16	41.7	20

## Data Availability

The original contributions presented in this study are included in the article. Further inquiries can be directed to the corresponding authors.
